# Do outcomes reported in randomised controlled trials of joint replacement surgery fulfil the OMERACT 2.0 Filter? A review of the 2008 and 2013 literature

**DOI:** 10.1186/s13643-017-0498-3

**Published:** 2017-05-30

**Authors:** Peter D. H. Wall, Bethan L. Richards, Andrew Sprowson, Rachelle Buchbinder, Jasvinder A. Singh

**Affiliations:** 1 Warwick Clinical Trials Unit, University of Warwick, Coventry, UK; 2 Institute of Rheumatology and Orthopaedics, Royal Prince Alfred Hospital, Sydney, Australia; 3 Sydney Medical School, University of Sydney, Sydney, Australia; 4 Department of Epidemiology and Preventive Medicine, School of Public Health & Preventive Medicine, Monash University, Frankston, VIC Australia; 5 Monash Department of Clinical Epidemiology, Cabrini Institute, Melbourne, VIC Australia; 6 Birmingham Veterans Affairs Medical Center and University of Alabama at Birmingham, Faculty Office Tower 805B, 510 20th Street S, Birmingham, AL 35294 USA; 7 Mayo Clinic School of Medicine, Rochester, MN USA

**Keywords:** Total joint arthroplasty, Systematic review, OMERACT filter, Core areas, Meta-analysis

## Abstract

**Background:**

It is not known, whether outcome reporting in trials of total joint arthroplasty in the recent years is adequate or not. Our objective was to assess whether outcomes reported in total joint replacement (TJR) trials fulfil the Outcome Measures in Rheumatology (OMERACT) Filter 2.0.

**Methods:**

We systematically reviewed all TJR trials in adults, published in English in 2008 or 2013. Searches were conducted in the Cochrane Central Register of Controlled Trials, MEDLINE, and EMBASE. Two authors independently applied the inclusion criteria for the studies, and any disagreement was resolved with a third review author. All outcome measures were abstracted using a pre-piloted standardised data extraction form and assessed for whether they mapped to one of the three OMERACT Filter 2.0 core areas: pathophysiological, life impact, and death.

**Results:**

From 1635 trials identified, we included 70 trials (30 in 2008 and 40 in 2013) meeting the eligibility criteria. Twenty-two (31%) trials reported the three essential OMERACT core areas. Among the 27 hip replacement surgery trials and 39 knee replacement surgery trials included, 11 hip (41%) and nine knee (23%) trials reported all three essential OMERACT core areas. The most common outcome domains/measures were pain (20/27, 74%) and function (23/27, 85%) in hip trials and pain (26/39, 67%) and function (27/39, 69%) in knee trials. Results were similar for shoulder and hand joint replacement trials.

**Conclusions:**

We identified significant gaps in the measurement of OMERACT core outcome areas in TJR trials, despite the majority reporting outcome domains of pain and function. An international consensus of key stakeholders is needed to develop a core domain set for reporting of TJR trials.

**Systematic review registration:**

PROSPERO CRD42014009216

**Electronic supplementary material:**

The online version of this article (doi:10.1186/s13643-017-0498-3) contains supplementary material, which is available to authorized users.

## Background

For a randomised controlled trial (RCT) to discern the true effect of an intervention, relevant and robust outcome measures must be chosen. A standardised set of outcome measures used across similar types of trials has the potential to increase their efficiency and value by enabling comparisons between trials and pooling of data, thereby providing more precise estimates of the treatment effect.

Twenty years ago the World Health Organization (WHO) and the International League of Associations for Rheumatology (ILAR) established a core set of outcomes for clinical trials in rheumatoid arthritis. This work originated from the Outcome Measures in Rheumatology (OMERACT) group that developed a framework and methodology (i.e. the OMERACT Filter), for the identification and validation of core outcome measurement sets for use in clinical trials, for any health condition [1]. The OMERACT group has gone on to develop successful core outcome measurement sets for other conditions including ankylosing spondylitis and gout, and the OMERACT Filter and methodology has been widely adopted internationally within the rheumatology community [1–3] and other disciplines [4–6].

Within the discipline of orthopaedic surgery, the development of a core outcome measurement set for trials involving patients with hip fractures is underway [7]. To our knowledge, there are currently no standardised or universally accepted core outcome measurement sets for clinical trials of joint replacement surgery. With over a million hip and knee joint replacements done each year in the USA alone [8], and the technology for joint replacement surgery evolving rapidly, there is a need for high-quality randomised controlled trials (RCTs). The use of standardised measures of outcome assessment in trials involving joint replacement will facilitate accurate and effective comparisons of new and existing joint replacement implants and techniques, as well as accurate and effective evaluation of the value of pre- and post-operative interventions.

In order to improve the reporting of relevant health outcome domains within joint replacement trials and develop a standard core set, a working group within OMERACT was established in 2008 and preliminary work was completed [9–11]. This work demonstrated the lack of well-validated outcome instruments in knee and hip clinical trials and identified the need to develop core outcome domains and a core outcome measurement set with the goal of harmonisation of outcome measures used in joint replacement clinical trials.

The OMERACT Filter 2.0 defines three “core areas” that should be measured within a clinical trial of any disease condition: death, life impact, and pathophysiological manifestations [1]; it also strongly recommends the measurement of resource utilisation. The OMERACT Filter 2.0 provides a roadmap, describing the steps to achieve a final core measurement set for clinical trials for a given condition. Firstly, it recommends relevant stakeholders start by identifying at least one “domain” within each of the core areas to formulate the “core domain set;” an additional file shows this in more detail (see Additional file 1). At least one applicable measurement instrument for each core domain is then identified to formulate a “core outcome measurement set.” Each measurement instrument must prove to be truthful (valid), discriminative, and reliable.

At the OMERACT-12 Meeting (2014), clinical and methodological experts in epidemiology, psychometrics, orthopaedics, and rheumatology along with patient partners interested in harmonising outcomes for people undergoing joint replacement surgery met as a working group. The ultimate aim of the group is to develop and reach international consensus on a core outcome measurement set for joint replacement surgery. In preparation for the meeting, we systematically examined the outcomes reported in all randomised controlled trials of joint replacement surgery published in 2008 and 2013. We found suboptimal reporting of primary outcomes in TJR trials as well as heterogeneity in the primary outcomes when reported [12]. In this paper, we report the extent to which the outcomes reported in the trials fulfil the OMERACT Filter 2.0 core areas of mortality, life impact, and pathophysiological manifestations, and the OMERACT Filter 2.0 strongly recommended area, resource use.

## Methods

We undertook the review in accordance with the Preferred Reporting Items for Systematic Reviews and Meta-Analyses (PRISMA) guidelines [13, 14]. A PRISMA checklist is provided as an additional file that shows this in more detail (see Additional file 2). The protocol for this review was registered with the International Prospective Register of Systematic Reviews (PROSPERO; Registration number: CRD42014009216).

We included all randomised or quasi-randomised (where allocation not strictly random) controlled trials investigating joint replacement surgery (defined as substitution of any joint surface with a prosthesis) in adult patients ≥18 years published in either 2008 or 2013. We chose 2 years only (2008 and 2013) for our study for two reasons: we anticipated that a 2-year data including a recent year would provide us with a reasonable sample size for our main study to assess consistency with OMERACT filter 2.0 [1]; and a secondary objective was to assess study quality and outcome reporting over time (2008 to 2013) and due to feasibility issues, since we expected >100 studies per year to be eligible, limited resources prohibited a review of 6-year trial data (reported in a separate manuscript ) [12]. We excluded trials investigating spinal joint replacement surgery and those trials where the intervention of interest was not part of the intraoperative insertion of joint replacement prosthesis, for example, trials investigating pre-operative education, peri-operative analgesia, or post-operative care.

The comparator could include another type of joint implant, surgical placebo or sham, usual care, physical therapy, or other active treatments. Trials were included if at least one outcome had been reported. Only trials published in English as full articles or available as full trial report were included.

We searched the Cochrane Central Register of Controlled Trials, MEDLINE, EMBASE, and hand searched reference lists of relevant articles for randomised or quasi-randomised controlled trials on 20 March 2014. We limited the search to publications in 2008 and 2013, in order to capture recent trials. The search strategy used for MEDLINE is provided as an additional file and shows this in more detail (see Additional file 3).

Two authors (BR and PW) independently assessed the search results based on the title and abstract, and the full texts of all potentially eligible studies were then assessed to identify studies that fulfilled inclusion criteria. Any disagreement in study selection was resolved by consensus or by discussion with a third review author (RB).

Trial details were extracted for each trial including the first author, year of publication, and interventions. Additional details including number of participants, year of recruitment, study duration, and sample size were also extracted but are reported in a separate manuscript [12].

We extracted all outcome measures using a standardised data extraction form. Outcome measures were then grouped according to outcome domains and then grouped according to the three OMERACT core areas, pathophysiological, life impact, and death or the recommended area, resource use. Joint-specific multidimensional outcome measures were broken down into constituent outcome domains and then grouped according to the four OMERACT core areas. The data was then aggregated and reported using simple summary statistics.

## Results

**Fig. 1:**
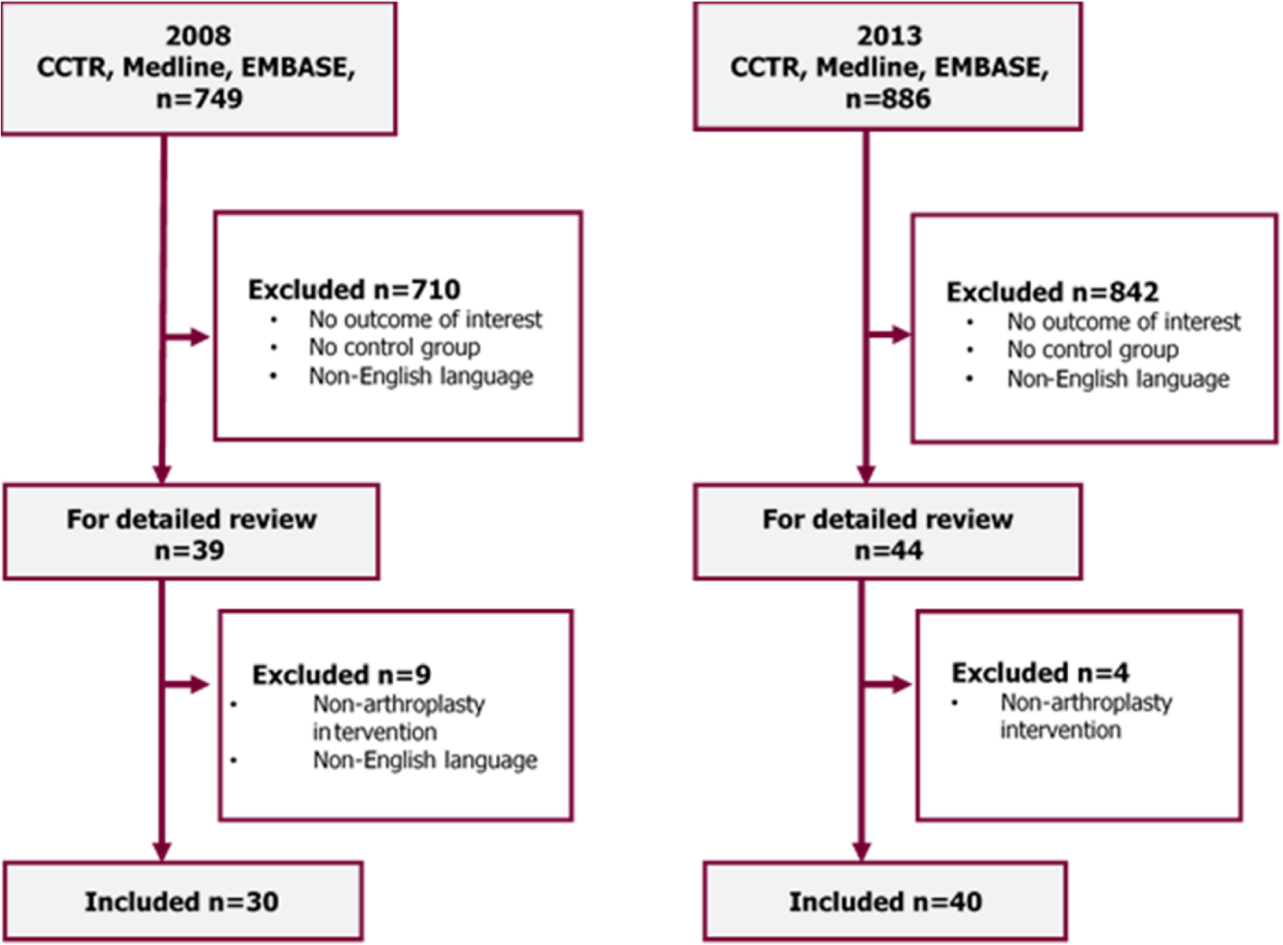
We identified 1635 potential studies  from the initial searches after de-duplication (41 duplicates in 2008 and 60 duplicates in 2013 were removed). Seventy trials, 30 published in 2008 and 40 published in 2013 met the eligibility criteria

There were a total of 1635 potential studies identified from the initial searches after de-duplication (41 duplicates in 2008 and 60 duplicates in 2013), and 70 trials (30 published in 2008 and 40 published in 2013) met the eligibility criteria and were included in the review (Fig. 1). Screening of titles/abstracts was done over 3 weeks, data abstraction over the next 4–6 weeks and data analyses for the 4 weeks after that. No published trials of joint replacement involving the foot, ankle, or elbow were identified. There were 27 trials for hip, 39 trials for knee, three trials for shoulder, and one trial for replacement surgery of the small joints (Table 1). The inter-rater agreement was 86% for 2008 and 93% for 2013 initial abstractions. One hundred percent consensus was reached by discussion and with involvement of a third reviewer. There were 13 joint-specific multidimensional outcome tools reported; all of which measured outcome domains of both pain and function (Table 2). Nine (69%) of the joint-specific multidimensional outcome tools were patient reported.

**Table 1:** Studies of hip and knee arthroplasty from 2008 to 2013

Author	Joint	Comparators
Garcia-Rey 2008 [20]	Hip	Ultrahigh molecular weight polyethylene liner THR vs. highly cross-linked polyethylene liner THR
Glyn-Jones 2008a [21]	Hip	Highly cross-linked polyethylene liner THR vs. ultrahigh molecular weight polyethylene liner THR
Glyn-Jones 2008b [22]	Hip	Highly cross-linked polyethylene liner THR vs. ultrahigh molecular weight polyethylene liner THR
Hamadouche 2008 [23]	Hip	Polished femoral stem THR vs. matte femoral stem THR
Lachiewicz 2008 [24]	Hip	Polished femoral stem THR vs. pre-coated femoral stem THR
Macaulay 2008 [25]	Hip	Hemiarthroplasty vs. THR
Meneghini 2008 [26]	Hip	Two incision minimally invasive THR vs. mini-posterior approach THR vs. mini-anterolateral approach THR
Mouzopoulos 2008 [27]	Hip	Hemiarthroplasty vs. THR vs. internal fixation
Pagnano 2008 [28]	Hip	Mini-incision THR vs. two incision THR
Pitto 2008 [29]	Hip	Polyethylene liner THR ceramic liner THR
Barrett 2013 [30]	Hip	Direct anterior approach THR vs. posterolateral approach THR
Bjorgul 2013 [31]	Hip	Metal-on-metal bearing THR vs. metal-on-polyethylene bearing THR vs. ceramic-on-polyethylene bearing THR
Cadossi 2013 [32]	Hip	Hemiarthroplasty vs. polycarbonateurethane acetabular component THR
Desmarchelier 2013 [33]	Hip	Metal-on-metal bearing THR vs. ceramic-on-ceramic bearing THR
Greidanus 2013 [34]	Hip	Minimally invasive anterolateral approach THR vs. minimally invasive direct lateral approach THR vs. minimally invasive posterolateral approach THR
Hedbeck 2013 [35]	Hip	Cemented hemiarthroplasty vs. internal fixation
Inngul 2013 [36]	Hip	Unipolar hemiarthroplasty vs. bipolar hemiarthroplasty
Kim 2013 [37]	Hip	Alumina-on-alumina ceramic bearing THR vs. alumina on highly cross-linked polyethylene bearing THR
Landgraeber 2013 [38]	Hip	Minimally invasive THR vs. conventional THR
Munzinger 2013 [39]	Hip	Titanium plasma-sprayed cup THR vs. titanium plasma-sprayed cup with additional hydroxyapatite coating THR
Naudie 2013 [40]	Hip	Sintered bead porous surface shell THR vs. titanium anatomic porous surface THR
Penny 2013 [41]	Hip	Standard THR vs. large head THR vs. resurfacing hip replacement
Smolders 2013 [42]	Hip	Resurfacing hip replacement vs. THR
Stiehler 2013 [43]	Hip	Navigated hip resurfacing vs. conventional hip resurfacing
Venditolli 2013 [44]	Hip	Alumina on alumina vs. metal-on-polyethylene THR
Vidovic 2013 [45]	Hip	Cemented hemiarthroplasty vs. cementless hemiarthroplasty
Zagra 2013 [46]	Hip	28 vs 36 vs. 42 mm bearing THR
Breugem 2008 [47]	Knee	Fixed bearing TKR vs. mobile bearing TKR
Chaudhary 2008 [48]	Knee	Posterior cruciate stabilising TKR vs. posterior cruciate-retaining TKR
Dutton 2008 [49]	Knee	Computer-assisted minimally invasive TKR vs. conventional TKR
Findlay 2008 [50]	Knee	Cemented TKR vs. uncemented TKR
Hall 2008 [51]	Knee	Single radius of curvature femoral component TKR vs. multi-radius of curvature femoral component TKR
Han 2008 [52]	Knee	Minimally invasive TKR vs. conventional TKR
Hansson 2008 [53]	Knee	HA-coated TKR vs. Not HA-coated TKR
Harato 2008 [54]	Knee	Posterior cruciate-retaining TKR vs. Posterior cruciate substituting TKR
Karachalios 2008 [55]	Knee	Mini-mid vastus approach TKR standard approach TKR
Ladermann 2008 [56]	Knee	Fixed bearing TKR vs. mobile bearing TKR
Lionberger 2008 [57]	Knee	Electromagnetic navigation TKR vs. infrared navigation TKR
Lozano 2008 [58]	Knee	Extramedullary tibial guide TKR vs. intramedullary tibial guide TKR
Luring 2008 [59]	Knee	Navigated TKR vs. minimally invasive TKR vs. conventional TKR
Lutzner 2008 [60]	Knee	Navigated TKR vs. conventional TKR
Nutton 2008 [61]	Knee	Standard Nexgen TKR vs. high flexion Nexgen TKR
Oberst 2008 [62]	Knee	Navigated TKR vs. conventional TKR
Smith 2008 [63]	Knee	Patellar resurfacing TKR vs. no patellar resurfacing TKR
Therbo 2008 [64]	Knee	HA coated tibial component TKR vs. no HA on tibial component TKR
Wylde 2008 [65]	Knee	Fixed bearing TKR vs. mobile bearing TKR
Aggarwal 2013 [66]	Knee	Fixed bearing TKR vs. mobile bearing TKR
Breeman 2013 [67]	Knee	Mobile bearing TKR vs. fixed bearing TKR
Chareancholvanich 2013 [68]	Knee	Patient-specific cutting guide TKR vs. conventional instrumentation TKR
Dennis 2013 [69]	Knee	High flexion TKR vs. standard device TKR
Fischer 2013 [70]	Knee	High flexion TKR vs. standard device TKR
Hamilton DF 2013a [71]	Knee	Triathlon TKR vs. Kinemax TKR
Hamilton DF 2013b [72]	Knee	Triathlon TKR vs. Kinemax TKR
Hamilton WG 2013 [73]	Knee	Patient-specific instrumentation TKR vs. traditional instrumentation TKR
Jarvis 2013 [74]	Knee	Standard parapatellar approach TKR vs. mini-parapatellar approach TKR
Joseph 2013 [75]	Knee	Computer navigation TKR vs. no computer navigation TKR
Jung 2013 [76]	Knee	Intramedullary alignment TKR vs. extra-medullary alignment TKR
Nieuwenhuijse 2013 [77]	Knee	LPS-flex mobile TKR vs. LPS-flex-fixed TKR vs. LPS-fixed TKR vs. LPS mobile TKR
Nishizawa 2013 [78]	Knee	Cruciate-retaining TKR vs. posterior stabilised TKR
Pandit 2013 [79]	Knee	Cemented unicompartmental knee replacement vs. cementless unicompartmental knee replacement
Radetzki 2013 [80]	Knee	High-flex NexGen LPS flex mobile bearing TKR vs. NexGen LPS TKR
Roh 2013 [81]	Knee	Patient-specific instruments TKR vs. conventional instruments TKR
Song 2013 [82]	Knee	Robotic-assisted TKR vs. conventional TKR instruments
Umrani 2013 [83]	Knee	Patellar eversion TKR vs. no patellar eversion TKR
Wegrzyn 2013 [84]	Knee	Mini-subvastus approach TKR vs. medial parapatellar approach TKR
Yim 2013 [85]	Knee	Robot-assisted classical alignment TKR vs. robot-assisted anatomical alignment TKR
Fialka 2008 [86]	Shoulder	Has shoulder hemiarthroplasty vs. epoca shoulder hemiarthroplasty
Soliman 2013 [87]	Shoulder	Hemiarthroplasty and tenodesis of the long head of the biceps vs. hemiarthroplasty without tenodesis of the long head of the biceps
Lapner 2013 [88]	Shoulder	Tuberosity osteotomy shoulder replacement vs. subscapularis peel shoulder replacement
Hansen 2013 [89]	Hand	Cemented vs. uncemented cups in total trapeziometacarpal joint prostheses

*TKR* total knee replacement, *THR* total hip replacement

**Table 2:** Constituent outcomes for multidimensional joint-specific outcome tools

Composite joint-specific outcome tool	Proportion of eligible trials reporting *n* (%)	Constituent outcomes measured
Merle D’Aubigné and Postel Score (MDPS)	3/27 (11)	Pain, function, ROM
Oxford Hip Score (OHS)	2/27 (7)	Pain, function
Harris Hip Score (HHS)	15/27 (56)	Pain, function, ROM
Hip disability and Osteoarthritis outcomes score (HOOS)	1/27 (4)	Pain, function, hip-related quality of life
Hospital for Special Surgery (HSS) Knee Score	4/39 (10)	Pain, function, ROM, knee stability, knee alignment (not using radiographs)
Knee Society Clinical Rating System (KSS)	16/39 (41)	Pain, function, ROM, knee stability, knee alignment (not using radiographs)
Knee injury and Osteoarthritis Outcome Score (KOOS)	3/39 (8)	Pain, function, knee-related quality of life
Oxford Knee Score (OKS)	6/39 (15)	Pain, function
Western Ontario and McMaster Universities Arthritis Index (WOMAC)	13/66 (20)	Pain, function, stiffness
Western Ontario Osteoarthritis of the Shoulder (WOOS)	1/3 (33)	Pain, function, shoulder-related quality of life
American Shoulder and Elbow Surgeons (ASES)	1/3 (33)	Pain, function, activity levels
Constant Score	1/3 (33)	Pain, strength, activity levels, ROM
Disabilities of the Arm, Shoulder and Hand (DASH) Score	1/4 (25)	Pain, function, strength, stiffness, hand-related quality of life

A mean of six outcome domains were reported per trial. Twenty-two (31%) trials reported outcome domains/measures in all three of the essential OMERACT core areas (pathophysiological, life impact, and death), and 21 (30%) trials reported outcome domains/measures in the recommended area of resource utilisation.

### Hip replacement trial outcome domains

Twenty-seven trials of hip replacement surgery were included (10 published in 2008 and 17 published in 2013) (Table 3). Eighteen unique outcome measures were identified with a mean of six outcome measures per trial. Eleven (41%) trials reported an outcome domain/measure within all three of the essential OMERACT core areas. The most common outcome domains/measures reported were pain (20/27, 74%) and function (23/27, 85%).

**Table 3:** Hip RCT outcomes and their mapping to the three core and one optional OMERACT areas/domains

Authors	Pathophysiological							Life impact					Death	All 3 core areas reported	Resource use/economic impact^a^				
	Pain	Stiffness	ROM	AE	Blood loss	RSA	RMAL	Satisfaction	Hip QoL	General QoL	Function	Activity levels	Mortality		LoI	Surgery time	LoS	Reop/readmit	Revision rate
Garcia-Rey 2008 [20]	√		√				√				√								
Glyn-Jones 2008a [21]	√					√	√				√								
Glyn-Jones 2008b [22]				√		√													
Hamadouche 2008 [23]	√		√	√			√				√		√	√					√
Lachiewicz 2008 [24]	√		√	√			√				√								√
Macaulay 2008 [25]	√	√	√	√						√	√		√	√					
Meneghini 2008 [26]				√							√								
Mouzopoulos 2008 [27]	√		√								√		√	√					
Pagnano 2008 [28]				√						√	√	√							
Pitto 2008 [29]	√		√				√				√								
Barrett 2013 [30]	√		√	√	√		√		√		√				√	√	√		
Bjorgul 2013 [31]	√		√		√		√				√		√	√		√			√
Cadossi 2013 [32]	√		√	√	√		√				√		√	√		√	√		√
Desmarchelier 2013 [33]	√		√	√			√	√			√	√	√	√					√
Greidanus 2013 [34]	√	√		√			√	√		√	√								
Hedbeck 2013 [35]							√			√	√		√	√				√	
Inngul 2013 [36]	√		√	√			√			√	√		√	√					
Kim 2013 [37]	√	√	√				√				√	√	√	√					
Landgraeber 2013 [38]	√	√	√	√	√		√				√		√	√	√	√			
Munzinger 2013 [39]						√													
Naudie 2013 [40]	√	√	√			√				√	√		√	√					
Penny 2013 [41]	√	√	√	√	√					√	√	√			√	√	√		
Smolders 2013 [42]							√						√						
Stiehler 2013 [43]	√	√	√				√			√	√								
Venditolli 2013 [44]							√											√	√
Vidovic 2013 [45]	√		√	√			√				√					√	√		
Zagra 2013 [46]	√		√								√								

11 RCTs had all three core areas reported

*AE* adverse events, *RSA* radiosteriometric analysis, *RMAL* radiographs to measure alignment or loosening, *Satis* satisfaction, *QoL* quality of life, *LoI* length of incision, *LoS* length of stay

^a^Area/domain recommended, but not a core area

Seven unique outcome domains/measures mapped to core area pathophysiological, five mapped to life impact, five mapped to resource use and one mapped to death. Core area pathophysiological was represented most frequently with 86 instances of mapping to this area.

### Knee replacement trial outcome domains

Thirty-nine trials of knee replacement surgery were included (19 published in 2008 and 20 in 2013) (Table 4). Twenty-one individual outcome domains/measures were identified with a mean of six per trial. Nine (23%) trials reported an outcome domain/measure within all three of the essential OMERACT core areas. The most common outcome domains/measures were pain (26/39, 67%) and function (27/39, 69%). Nine outcome domains mapped to pathophysiological, five mapped to life impact, six mapped to resource use and one outcome mapped to death. Core area pathophysiological was represented most frequently, with 150 instances of mapping to this area.

**Table 4:** Knee study outcomes and their mapping to three core and one optional OMERACT areas/domains

Authors	Pathophysiological									Life impact					Death	All 3 core areas reported	Resource use/economic impact^a^					
	Pain	Stiffness	Knee stability	Knee alignment (clinical)	ROM	AE	Blood loss	RSA	RMAL	Satis	Knee QoL	General QoL	Function	Activity levels	Mortality		LoI	Surgery time	No. trays	LoS	Reop/readmit	Cost per patient
Breugem 2008 [47]	√		√	√	√	√			√			√	√		√	√						
Chaudhary 2008 [48]	√	√			√	√						√	√		√	√					√	
Dutton 2008 [49]	√		√	√	√	√	√					√	√					√				
Findlay 2008 [50]	√		√	√	√	√			√				√									
Hall 2008 [51]	√		√	√	√								√									
Han 2008 [52]					√	√	√		√				√					√				
Hansson 2008 [53]	√		√	√				√					√									
Harato 2008 [54]	√	√	√	√	√	√			√			√	√		√	√						
Karachalios 2008 [55]	√		√	√	√	√	√		√				√					√				
Ladermann 2008 [56]	√		√	√	√	√						√	√		√	√						
Lionberger 2008 [57]									√													
Lozano 2008 [58]									√									√				
Luring 2008 [59]	√	√	√	√	√	√	√		√				√				√	√				
Lutzner 2008 [60]						√			√						√							
Nutton 2008 [61]	√	√	√	√	√							√	√									
Oberst 2008 [62]									√													
Smith 2008 [63]	√		√	√	√	√			√	√			√									
Therbo 2008 [64]	√		√	√	√			√					√									
Wylde 2008 [65]	√	√				√					√	√	√		√	√						
Aggarwal 2013 [66]	√		√	√	√	√			√	√			√		√	√						
Breeman 2013 [67]	√					√						√	√					√		√	√	√
Chareancholvanich 2013 [68]						√	√		√								√	√		√		
Dennis 2013 [69]	√		√	√	√						√		√									
Fischer 2013 [70]	√		√	√	√							√	√									
Hamilton DF 2013a [71]												√										
Hamilton DF 2013b [72]	√	√							√				√									
Hamilton WG 2013 [73]									√									√	√			
Jarvis 2013 [74]													√									
Joseph 2013 [75]					√				√													
Jung 2013 [76]									√													
Nieuwenhuijse 2013 [77]	√		√	√	√			√	√				√		√	√						
Nishizawa 2013 [78]									√													
Pandit 2013 [79]	√		√	√	√				√				√	√	√	√						
Radetzki 2013 [80]	√		√	√	√				√				√		√	√						
Roh 2013 [81]									√													
Song 2013 [82]	√	√	√	√	√	√			√				√					√				
Umrani 2013 [83]	√		√	√	√							√	√									
Wergrzyn 2013 [84]	√										√	√	√	√								
Yim 2013 [85]	√	√	√	√	√				√				√									

9 RCTs had all the three core areas reported

^a^Area/domain recommended, but not a core area

### Shoulder replacement trial outcome domains

There were three (4%) trials of shoulder replacement surgery; an additional file shows this in more detail (see Additional file 4). Outcome domains/measures of pain, strength, and activity levels were reported in all three trials. Seven outcome domains mapped to pathophysiological, three mapped to life impact, and one outcome domain mapped to death. Core area pathophysiological was represented most frequently with 12 instances of mapping to this area.

### Hand joint replacement outcome domains

There was one (1%) trial involving replacement of the small joints of the hand reporting six individual outcome domains/measures with four mapping to pathophysiological and two to life impact core areas. An additional file shows this in more detail (see Additional file 4).

## Discussion

The purpose of this systematic review was to examine and highlight inconsistencies in reporting of joint replacement trials and make recommendations for future studies in the area. This systematic review has highlighted that there are significant gaps in the measurement of OMERACT core outcome areas in joint replacement trials. Less than a third (31%) of trials captured outcome domains/measures within all three essential OMERACT core areas. The majority of joint replacement trials (but not all) did, however, capture outcome domains/measures of pain (71%) and function (77%). This finding is in keeping with the principles and primary indications for joint replacement surgery, which are to relieve pain and improve function. All of the joint-specific multidimensional outcome tools included in the trials capture both pain and function, which is a reflection that these measures are well established and accepted by the orthopaedic community for monitoring outcomes after joint replacement surgery [15].

All trials captured domains within the core area of pathophysiological manifestations, with many trials reporting surrogate outcome domains such as radiosteriometric analysis (RSA) and plain radiographs to assess implant loosening. RSA uses x-rays to determine the implant position and is a well-validated tool for measuring the movement of implants following joint replacement surgery. RSA requires specialist equipment and training to use and therefore really only has a role in early/short-term clinical evaluation of joint replacements. The correlation between movement detected on RSA and longer term clinically meaningful implant failure is not well documented or validated [16]. It is not surprising that the OMERACT filter 2.0 framework specifies both pathophysiological manifestations and life impact (such as pain, function, mobility, quality of life) as two of the three core areas for any disease construct. In our example, filter 2.0 indicates that it is just as important (if not more) to know the true clinical impact of a difference in implant positioning between interventions, i.e. implant failure/revision and pain, function, quality of life (impact on the patient) as is knowing the exact positioning of the implant (e.g. by RSA).

Measurement of mortality is one of the three core areas of the OMERACT Filter 2.0, but was reported in only 36% of the trials reviewed. In addition, none of the trials reported whether or not mortality was considered attributable to the interventions under study or underlying condition/s. Measurement and reporting of 7-, 30- and 90-day mortality, or mortality during the trial (3 or 6 or 12 months) could capture potential intervention-related versus unrelated deaths and be supplemented with a case by case review to determine the cause of death. For joint replacement, which is usually an elective procedure, mortality is rare, but unexpected. Therefore, mortality reporting is very important. As in any clinical trial, study subject mortality is always known to the investigator and its reporting is quite simple, i.e. “there were no deaths in this trial,” and or adding a row with zeros (or the number applicable) to the table showing adverse events of each intervention being compared.

We also found that less than a third (31%) of trials captured the OMERACT recommended area of resource utilisation. Without comprehensive data about resource utilisation, it is difficult to determine the true comparative effectiveness (and cost-effectiveness) of one type of joint replacement compared to another. A potential reason for this may be a lack of appropriate outcome measures or a lack of consensus as to which outcome measure/s to use. Joint replacement is typically an elective surgery, and therefore, in principle, resource utilisation is pertinent and appropriate to capture from both the individual’s and system’s perspective. Outcome tools would need to be identified which could capture the individual initial costs of surgery and follow-up hospital visits but also any additional costs incurred as a result of further surgery or its complications.

One of the limitations of this review is that we only included two snapshots of joint replacement research trials, i.e. trial results published in 2008 and 2013. Our results may therefore not be truly representative of periods just before, between and after these dates. On the other hand, there is no reason to suspect that outcomes/measures and trial reporting would differ significantly different in other years.

Successful adoption of the original OMERACT filter [17] for validation of measures has led to the successful development and implementation of core domain sets and core measurement sets for various rheumatic and non-rheumatic diseases [1, 4–6, 18]. An updated version, OMERACT filter 2.0, is based on the WHO framework [1]. OMERACT filter 2.0 provides a practical framework to develop and validate domains and measures for any health condition. A pragmatic approach is to use a data-driven, consensus-based process with multi-stakeholder involvement to define a minimum measurement set for all joint replacement trials. In line with the OMERACT working group’s future agenda for achieving an international consensus-based core domain set for joint replacement trials, and building upon the findings of this review, we have derived a preliminary core domain set for joint replacement clinical trials based on the OMERACT filter 2.0 and multi-stakeholder consensus. The joint replacement clinical trial core domain set includes six core domains: pain, function, patient satisfaction, revision, adverse events, and death [19].

## Conclusions

In conclusion, this systematic review provides insights into the outcome areas/domains being used and reported in contemporary joint replacement RCTs and highlights the gaps in this area. The minimum standard of outcome reporting within joint replacement trials needs improvement. The OMERACT Filter [1] provides a well-established methodology for improving this, i.e. providing guidance and methods for developing a core outcome measurement set. RCTs are expensive time-consuming studies. As researchers, we have a duty to patients to extract as much clinically useful information as possible. The development of a core outcome measurement set for joint replacement trials would undoubtedly help to strengthen both the design and subsequent reporting of results in much the same way as it has within rheumatology clinical trials, and hopefully advance the field at an accelerated pace, by allowing comparisons across trials and standard meta-analyses.

## Additional files

Additional file 1: OMERACT conceptual framework of Core Areas for outcome measurement in the setting of healthcare intervention studies—reproduced from M. Boers et al. 2013J Clinical Epidemiology. Description of data: This file shows the schema of OMERACT conceptual framework of Core Areas for outcome measurement in the setting of healthcare intervention studies, which is based on the World Health Organization’s (WHO) International Classification of Functioning, Disability and Health (ICF) framework and forms the basis of OMERACT Filter 2.0. (DOCX 142 kb)

Additional file 2: PRISMA 2009 Checklist. Description of data: This file shows the PRISMA checklist for this systematic review. (DOCX 27 kb)

Additional file 3: Example search strategy for MEDLINE. Description of data: This file shows an example of a search strategy for MEDLINE database. (DOCX 47 kb)

Additional file 4: Shoulder and Hand Study Outcomes. Description of data: This file shows the included studies for shoulder and hand joint replacement and which of the core areas/domains of OMERACT Filter 2.0 were presented as outcomes in these trials. (DOCX 67 kb)

## Abbreviations

AE: Adverse events
CCTs: Controlled clinical trials
LoI: Length of incision
LoS: Length of stay
OMERACT: Outcome Measures in Rheumatology
QoL: Quality of life
RCT: Randomised controlled trial
RCTs: Randomised controlled trials
RMAL: Radiographs to measure alignment or loosening
RSA: Radiosteriometric analysis
Satis: Satisfaction
THR: Total hip replacement
TJA: Total joint arthroplasty
TKR: Total knee replacement
UAB: University of Alabama at Birmingham
WG: Working group
WHO: World Health Organization

## References

[1] Boers M, Kirwan JR, Wells G, Beaton D, Gossec L, d’Agostino MA (2014). Developing core outcome measurement sets for clinical trials: OMERACT filter 2.0. J Clin Epidemiol.

[2] Felson DT, Anderson JJ, Boers M, Bombardier C, Furst D, Goldsmith C (1995). American College of Rheumatology. Preliminary definition of improvement in rheumatoid arthritis. Arthritis Rheum.

[3] Schumacher HR, Taylor W, Edwards L, Grainger R, Schlesinger N, Dalbeth N (2009). Outcome domains for studies of acute and chronic gout. J Rheumatol.

[4] Chiarotto A, Terwee CB, Deyo RA, Boers M, Lin CW, Buchbinder R (2014). A core outcome set for clinical trials on non-specific low back pain: study protocol for the development of a core domain set. Trials.

[5] Hall NJ, Kapadia MZ, Eaton S, Chan WW, Nickel C, Pierro A (2015). Outcome reporting in randomised controlled trials and meta-analyses of appendicitis treatments in children: a systematic review. Trials.

[6] Schmitt J, Spuls PI, Thomas KS, Simpson E, Furue M, Deckert S (2014). The Harmonising Outcome Measures for Eczema (HOME) statement to assess clinical signs of atopic eczema in trials. J Allergy Clin Immunol.

[7] Haywood KL, Griffin XL, Achten J, Costa ML (2014). Developing a core outcome set for hip fracture trials. Bone Joint J.

[8] National Institue of Arthritis and Musculoskeletal and Skin Diseases (2014). Joint Replacement Surgery: Health Information Basics For You And Your Family.

[9] Riddle DL, Stratford PW, Bowman DH (2008). Findings of extensive variation in the types of outcome measures used in hip and knee replacement clinical trials: a systematic review. Arthritis Rheum.

[10] Riddle DL, Stratford PW, Singh JA, Strand CV (2009). Variation in outcome measures in hip and knee arthroplasty clinical trials: a proposed approach to achieving consensus. J Rheumatol.

[11] Gossec L, Paternotte S, Bingham CO, Clegg DO, Coste P, Conaghan PG (2011). OARSI/OMERACT initiative to define states of severity and indication for joint replacement in hip and knee osteoarthritis. An OMERACT 10 Special Interest Group. J Rheumatol.

[12] Richards B, Wall PDH, Sprowson AP, Singh JA, Buchbinder R. Outcome measures used in arthroplasty trials: Systematic review of the 2008 and 2013 literature. J Rheumatol. 2017. doi:10.3899/jrheum.161477. [Epub ahead of print]. PMID:28507180. 10.3899/jrheum.161477 28507180

[13] Liberati A, Altman DG, Tetzlaff J, Mulrow C, Gotzsche PC, Ioannidis JP (2009). The PRISMA statement for reporting systematic reviews and meta-analyses of studies that evaluate health care interventions: explanation and elaboration. PLoS Med.

[14] Moher D, Liberati A, Tetzlaff J, Altman DG (2009). Preferred reporting items for systematic reviews and meta-analyses: the PRISMA statement. PLoS Med.

[15] Black N (2013). Patient reported outcome measures could help transform healthcare. BMJ.

[16] Karrholm J, Gill RH, Valstar ER (2006). The history and future of radiostereometric analysis. Clin Orthop Relat Res.

[17] Boers M, Brooks P, Strand CV, Tugwell P (1998). The OMERACT filter for Outcome Measures in Rheumatology. J Rheumatol.

[18] Felson DT, Anderson JJ, Boers M, Bombardier C, Chernoff M, Fried B (1993). The American College of Rheumatology preliminary core set of disease activity measures for rheumatoid arthritis clinical trials. The Committee on Outcome Measures in Rheumatoid Arthritis Clinical Trials. Arthritis Rheum.

[19] Singh JA, Dohm M, Sprowson AP, Wall PD, Richards BL, Gossec L, et al. Outcome Domains and Measures in Total Joint Replacement Clinical Trials: Can We Harmonize Them? An OMERACT Collaborative Initiative. J Rheumatol. 2015;42(12):2496–502. 10.3899/jrheum.141201 25834208

[20] Garcia-Rey E, Garcia-Cimbrelo E, Cruz-Pardos A, Ortega-Chamarro J (2008). New polyethylenes in total hip replacement: a prospective, comparative clinical study of two types of liner. J Bone Joint Surg Br.

[21] Glyn-Jones S, Isaac S, Hauptfleisch J, McLardy-Smith P, Murray DW, Gill HS (2008). Does highly cross-linked polyethylene wear less than conventional polyethylene in total hip arthroplasty? A double-blind, randomized, and controlled trial using roentgen stereophotogrammetric analysis. J Arthroplasty.

[22] Glyn-Jones S, McLardy-Smith P, Gill HS, Murray DW (2008). The creep and wear of highly cross-linked polyethylene: a three-year randomised, controlled trial using radiostereometric analysis. J Bone Joint Surg.

[23] Hamadouche M, Baque F, Lefevre N, Kerboull M (2008). Minimum 10-year survival of Kerboull cemented stems according to surface finish. Clin Orthop Relat Res.

[24] Lachiewicz PF, Kelley SS, Soileau ES (2008). Survival of polished compared with precoated roughened cemented femoral components: a prospective, randomized study. J Bone Joint Surg Ser A.

[25] Macaulay W, Nellans KW, Garvin KL, Iorio R, Healy WL, Rosenwasser MP (2008). Prospective randomized clinical trial comparing hemiarthroplasty to total hip arthroplasty in the treatment of displaced femoral neck fractures. Winner of the Dorr Award. J Arthroplasty.

[26] Meneghini RM, Smits SA, Swinford RR, Bahamonde RE (2008). A randomized, prospective study of 3 minimally invasive surgical approaches in total hip arthroplasty. comprehensive gait analysis. J Arthroplasty.

[27] Mouzopoulos G, Stamatakos M, Arabatzi H, Vasiliadis G, Batanis G, Tsembeli A (2008). The four-year functional result after a displaced subcapital hip fracture treated with three different surgical options. Int Orthop.

[28] Pagnano MW, Trousdale RT, Meneghini RM, Hanssen AD (2008). Slower recovery after two-incision than mini-posterior-incision total hip arthroplasty: a randomized clinical trial. J Bone Joint Surg Ser A.

[29] Pitto RP, Bhargava A, Pandit S, Munro JT (2008). Retroacetabular stress-shielding in THA. Clin Orthop.

[30] Barrett WP, Turner SE, Leopold JP (2013). Prospective randomized study of direct anterior vs postero-lateral approach for total hip arthroplasty. J Arthroplasty.

[31] Bjorgul K, Novicoff WN, Andersen ST, Ahlund OR, Bunes A, Wiig M (2013). High rate of revision and a high incidence of radiolucent lines around Metasul metal-on-metal total hip replacements: results from a randomised controlled trial of three bearings after seven years. Bone Joint J.

[32] Cadossi M, Chiarello E, Savarino L, Tedesco G, Baldini N, Faldini C (2013). A comparison of hemiarthroplasty with a novel polycarbonate-urethane acetabular component for displaced intracapsular fractures of the femoral neck: a randomised controlled trial in elderly patients. [Erratum appears in Bone Joint J. 2013 Nov;95-B(11):1582]. Bone Joint J.

[33] Desmarchelier R, Viste A, Chouteau J, Lerat JL, Fessy MH (2013). Metasul vs cerasul bearings. A prospective, randomized study at 9 years. J Arthroplasty.

[34] Greidanus NV, Chihab S, Garbuz DS, Masri BA, Tanzer M, Gross AE (2013). Outcomes of minimally invasive anterolateral THA are not superior to those of minimally invasive direct lateral and posterolateral THA hip. Clin Orthop Relat Res.

[35] Hedbeck CJ, Inngul C, Blomfeldt R, Ponzer S, Tornkvist H, Enocson A (2013). Internal fixation versus cemented hemiarthroplasty for displaced femoral neck fractures in patients with severe cognitive dysfunction: a randomized controlled trial. J Orthop Trauma.

[36] Inngul C, Hedbeck CJ, Blomfeldt R, Lapidus G, Ponzer S, Enocson A (2013). Unipolar hemiarthroplasty versus bipolar hemiarthroplasty in patients with displaced femoral neck fractures. A four-year follow-up of a randomised controlled trial. Int Orthop.

[37] Kim YH, Park JW, Kulkarni SS (2013). A randomised prospective evaluation of ceramic-on-ceramic and ceramic-on-highly cross-linked polyethylene bearings in the same patients with primary cementless total hip arthroplasty. Int Orthop.

[38] Landgraeber S, Quitmann H, Guth S, Haversath M, Kowalczyk W, Kecskemethy A (2013). A prospective randomized peri- and post-operative comparison of the minimally invasive anterolateral approach versus the lateral approach. Orthop Rev.

[39] Munzinger U, Guggi T, Kaptein B, Persoon M, Valstar E, Cornelis Doets H (2013). A titanium plasma-sprayed cup with and without hydroxyapatite-coating: a randomised radiostereometric study of stability and osseointegration. Hip Int.

[40] Naudie DDR, Somerville L, Korczak A, Yuan X, McCalden RW, Holdsworth D (2013). A randomized trial comparing acetabular component fixation of two porous ingrowth surfaces using RSA. J Arthroplasty.

[41] Penny JO, Ovesen O, Varmarken J-E, Overgaard S (2013). Similar range of motion and function after resurfacing large-head or standard total hip arthroplasty. Acta Orthop.

[42] Smolders JMH, Pakvis DF, Hendrickx BW, Verdonschot N, van Susante JLC (2013). Periacetabular bone mineral density changes after resurfacing hip arthroplasty versus conventional total hip arthroplasty: a randomized controlled DEXA study. J Arthroplasty.

[43] Stiehler M, Goronzy J, Hartmann A, Krummenauer F, Gunther K-P (2013). The First SICOT Oral Presentation Award 2011: imageless computer-assisted femoral component positioning in hip resurfacing: a prospective randomised trial. Int Orthop.

[44] Vendittoli PA, Riviere C, Lavigne M, Lavoie P, Alghamdi A, Duval N (2013). Alumina on alumina versus metal on conventional polyethylene: a randomized clinical trial with 9 to 15 years follow-up. Acta Orthop Belg.

[45] Vidovic D, Matejcic A, Punda M, Ivica M, Tomljenovic M, Bekavac-Beslin M (2013). Periprosthetic bone loss following hemiarthroplasty: a comparison between cemented and cementless hip prosthesis. Injury.

[46] Zagra L, Anasetti F, Bianchi L, Licari V, Giacometti Ceroni R (2013). No difference in gait recovery after THA with different head diameters: a prospective randomized study. Clin Orthop Relat Res.

[47] Breugem SJ, Sierevelt IN, Schafroth MU, Blankevoort L, Schaap GR, van Dijk CN (2008). Less anterior knee pain with a mobile-bearing prosthesis compared with a fixed-bearing prosthesis. Clin Orthop Relat Res.

[48] Chaudhary R, Beaupre LA, Johnston DW (2008). Knee range of motion during the first two years after use of posterior cruciate-stabilizing or posterior cruciate-retaining total knee prostheses. A randomized clinical trial. J Bone Joint Surg.

[49] Dutton AQ, Yeo SJ, Yang KY, Lo NN, Chia KU, Chong HC (2008). Computer-assisted minimally invasive total knee arthroplasty compared with standard total knee arthroplasty. A prospective, randomized study. J Bone Joint Surg.

[50] Findlay IA, Bowman NK, Miles K, East DJ, Apthorp HD, Butler-Manuel A. The AGC total knee replacement-cemented versus cementless hydroxyapatite fixation. Proceedings of the International Symposium on Current Topics in Knee Arthroplasty: 13th - 15th June 2007, Marbella, Spain; Selected Scientific Papers. J Bone Joint Surg. July 2008; Volume 90-B, Issue SUPP II.

[51] Hall J, Copp SN, Adelson WS, D’Lima DD, Colwell CW (2008). Extensor mechanism function in single-radius vs multiradius femoral components for total knee arthroplasty. J Arthroplasty.

[52] Han I, Seong SC, Lee S, Yoo JH, Lee MC (2008). Simultaneous bilateral MIS-TKA results in faster functional recovery. Clin Orthop.

[53] Hansson U, Ryd L, Toksvig-Larsen S (2008). A randomised RSA study of peri-Apatite HA coating of a total knee prosthesis. Knee.

[54] Harato K, Bourne RB, Victor J, Snyder M, Hart J, Ries MD (2008). Midterm comparison of posterior cruciate-retaining versus -substituting total knee arthroplasty using the Genesis II prosthesis. A multicenter prospective randomized clinical trial. Knee.

[55] Karachalios T, Giotikas D, Roidis N, Poultsides L, Bargiotas K, Malizos KN (2008). Total knee replacement performed with either a mini-midvastus or a standard approach: a prospective randomised clinical and radiological trial. J Bone Joint Surg Ser B.

[56] Ladermann A, Lubbeke A, Stern R, Riand N, Fritschy D (2008). Fixed-bearing versus mobile-bearing total knee arthroplasty: a prospective randomised, clinical and radiological study with mid-term results at 7 years. Knee.

[57] Lionberger DR, Weise J, Ho DM, Haddad JL (2008). How does electromagnetic navigation stack up against infrared navigation in minimally invasive total knee arthroplasties?. J Arthroplasty.

[58] Lozano LM, Segur JM, Macule F, Nunez M, Torner P, Castillo F (2008). Intramedullary versus extramedullary tibial cutting guide in severely obese patients undergoing total knee replacement: a randomized study of 70 patients with Body Mass Index >35 kg/m2. Obes Surg.

[59] Luring C, Beckmann J, Haibock P, Perlick L, Grifka J, Tingart M (2008). Minimal invasive and computer assisted total knee replacement compared with the conventional technique: a prospective, randomised trial. Knee Surg Sports Traumatol Arthrosc.

[60] Lutzner J, Krummenauer F, Wolf C, Gunther KP, Kirschner S (2008). Computer-assisted and conventional total knee replacement: a comparative, prospective, randomised study with radiological and CT evaluation. J Bone Joint Surg Ser B.

[61] Nutton RW, van der Linden ML, Rowe PJ, Gaston P, Wade FA (2008). A prospective randomised double-blind study of functional outcome and range of flexion following total knee replacement with the NexGen standard and high flexion components. J Bone Joint Surg Br.

[62] Oberst M, Bertsch C, Konrad G, Lahm A, Holz U (2008). CT analysis after navigated versus conventional implantation of TKA. Arch Orthop Trauma Surg.

[63] Smith AJ, Wood DJ, Li MG (2008). Total knee replacement with and without patellar resurfacing: a prospective, randomised trial using the profix total knee system. J Bone Joint Surg Br.

[64] Therbo M, Lund B, Jensen KE, Schroder HM (2008). Effect of bioactive coating of the tibial component on migration pattern in uncemented total knee arthroplasty: a randomized RSA study of 14 knees presented according to new RSA-guidelines. J Orthop Traumatol.

[65] Wylde V, Learmonth I, Potter A, Bettinson K, Lingard E (2008). Patient-reported outcomes after fixed- versus mobile-bearing total knee replacement: a multi-centre randomised controlled trial using the Kinemax total knee replacement. [Erratum appears in J Bone Joint Surg Br. 2008 Nov;90(11):1534]. J Bone Joint Surg Br.

[66] Aggarwal AK, Agrawal A (2013). Mobile vs fixed-bearing total knee arthroplasty performed by a single surgeon. A 4- to 6.5-year randomized, prospective, controlled, double-blinded study. J Arthroplasty.

[67] Breeman S, Campbell MK, Dakin H, Fiddian N, Fitzpatrick R, Grant A (2013). Five-year results of a randomised controlled trial comparing mobile and fixed bearings in total knee replacement. Bone Joint J.

[68] Chareancholvanich K, Narkbunnam R, Pornrattanamaneewong C (2013). A prospective randomised controlled study of patient-specific cutting guides compared with conventional instrumentation in total knee replacement. Bone Joint J.

[69] Dennis DA, Heekin RD, Clark CR, Murphy JA, O’Dell TL, Dwyer KA (2013). Effect of implant design on knee flexion. J Arthroplasty.

[70] Fischer M, von Eisenhart-Rothe R, Simank HG (2013). Comparable short-term results seen with standard and high-flexion knee arthroplasty designs in European patients. J Orthop.

[71] Hamilton DF, Simpson A, Burnett R, Patton JT, Moran M, Clement ND (2013). Lengthening the moment arm of the patella confers enhanced extensor mechanism power following total knee arthroplasty. J Orthop Res.

[72] Hamilton DF, Clement ND, Burnett R, Patton JT, Moran M, Howie CR (2013). Do modern total knee replacements offer better value for money? A health economic analysis. Int Orthop.

[73] Hamilton WG, Parks NL, Saxena A (2013). Patient-specific instrumentation does not shorten surgical time: a prospective, randomized trial. J Arthroplasty.

[74] Jarvis SL, Johnson-Wo AK, Onstot BR, Bhowmik-Stoker M, Shrader MW, Jacofsky MC (2013). Differences between standard and minimally invasive parapatellar surgical approaches for total knee arthroplasty in the tasks of sitting and standing. J Knee Surg.

[75] Joseph J, Simpson PMS, Whitehouse SL, English HW, Donnelly WJ (2013). The use of navigation to achieve soft tissue balance in total knee arthroplasty—A randomised clinical study. Knee.

[76] Jung W, Chun C, Lee J, Ha J, Jeong JH (2013). The accuracy of the extramedullary and intramedullary femoral alignment system in total knee arthroplasty for varus osteoarthritic knee. Knee Surg Sports Traumatol Arthrosc.

[77] Nieuwenhuijse MJ, van der Voort P, Kaptein BL, van der Linden-van der Zwaag HM, Valstar ER, Nelissen RG (2013). Fixation of high-flexion total knee prostheses: five-year follow-up results of a four-arm randomized controlled clinical and roentgen stereophotogrammetric analysis study. J Bone Joint Surg.

[78] Nishizawa Y, Matsumoto T, Kubo S, Muratsu H, Matsushita T, Oka S (2013). The influence of patella height on soft tissue balance in cruciate-retaining and posterior-stabilised total knee arthroplasty. Int Orthop.

[79] Pandit H, Liddle AD, Kendrick BJ, Jenkins C, Price AJ, Gill HS (2013). Improved fixation in cementless unicompartmental knee replacement: five-year results of a randomized controlled trial. J Bone Joint Surg Am.

[80] Radetzki F, Wienke A, Mendel T, Gutteck N, Delank KS, Wohlrab D (2013). High flex total knee arthroplasty—a prospective, randomized study with results after 10 years. Acta Orthop Belg.

[81] Roh YW, Kim TW, Lee S, Seong SC, Lee MC (2013). Is TKA using patient-specific instruments comparable to conventional TKA? A randomized controlled study of one system knee. Clin Orthop Relat Res.

[82] Song EK, Seon JK, Yim JH, Netravali NA, Bargar WL (2013). Robotic-assisted TKA reduces postoperative alignment outliers and improves gap balance compared to conventional TKA knee. Clin Orthop Relat Res.

[83] Umrani SP, Cho KY, Kim KI (2013). Patellar eversion does not adversely affect quadriceps recovery following total knee arthroplasty. J Arthroplasty.

[84] Wegrzyn J, Parratte S, Coleman-Wood K, Kaufman KR, Pagnano MW (2013). The John Insall award: no benefit of minimally invasive TKA on gait and strength outcomes: a randomized controlled trial. Clin Orthop Relat Res.

[85] Yim J-H, Song E-K, Khan MS, Sun ZH, Seon J-K (2013). A comparison of classical and anatomical total knee alignment methods in robotic total knee arthroplasty: classical and anatomical knee alignment methods in TKA. J Arthroplasty.

[86] Fialka C, Stampfl P, Arbes S, Reuter P, Oberleitner G, Vecsei V (2008). Primary hemiarthroplasty in four-part fractures of the proximal humerus: Randomized trial of two different implant systems. J Shoulder Elbow Surg.

[87] Soliman OA, Koptan WMT (2013). Proximal humeral fractures treated with hemiarthroplasty: does tenodesis of the long head of the biceps improve results?. Injury.

[88] Lapner PLC, Sabri E, Rakhra K, Bell K, Athwal GS (2013). Healing rates and subscapularis fatty infiltration after lesser tuberosity osteotomy versus subscapularis peel for exposure during shoulder arthroplasty. J Shoulder Elbow Surg.

[89] Hansen TB, Stilling M (2013). Equally good fixation of cemented and uncemented cups in total trapeziometacarpal joint prostheses. Acta Orthop.

