# Supplementary material for: Do outcomes reported in randomised controlled trials of joint replacement surgery fulfil the OMERACT 2.0 Filter? A review of the 2008 and 2013 literature
Source: Syst Rev. 2017 May 30;6:106. doi: 10.1186/s13643-017-0498-3 (PMC5450048; doi:10.1186/s13643-017-0498-3)
Supplement: Supplementary file 1 — OMERACT conceptual framework of Core Areas for outcome measurement in the setting of healthcare intervention studies—reproduced from M. Boers et al. 2013J Clinical Epidemiology. Description of data: This file shows the schema of OMERACT conceptual framework of Core Areas for outcome measurement in the setting of healthcare intervention studies, which is based on the World Health Organization’s (WHO) International Classification of Functioning, Disability and Health (ICF) framework and forms the basis of OMERACT Filter 2.0. (DOCX 142 kb) [file 13643_2017_498_MOESM1_ESM.docx]

Additional File 1. OMERACT conceptual framework of Core Areas for outcome measurement in the setting of healthcare intervention studies – reproduced from M. Boers et al. 2013 J Clinical Epidemiology [[1](#_ENREF_1)]
