# Supplementary figures and images for: Do outcomes reported in randomised controlled trials of joint replacement surgery fulfil the OMERACT 2.0 Filter? A review of the 2008 and 2013 literature
Source: Syst Rev. 2017 May 30;6:106. doi: 10.1186/s13643-017-0498-3 (PMC5450048; doi:10.1186/s13643-017-0498-3)

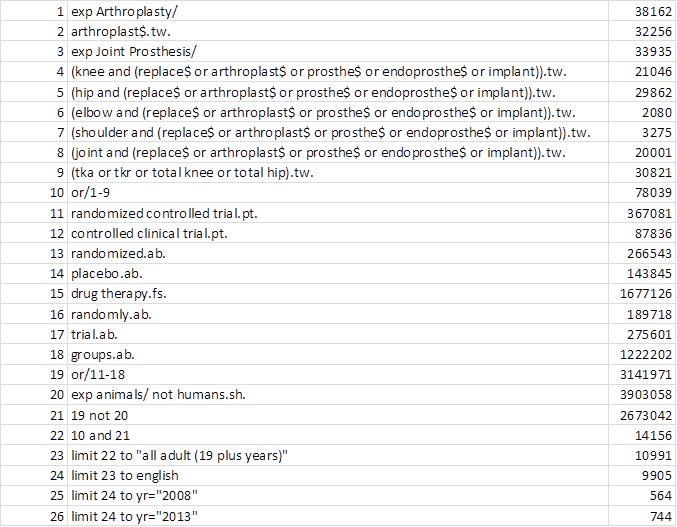


Additional File 3. Example search strategy for MEDLINE

Supplement: Supplementary file 3 — Example search strategy for MEDLINE. Description of data: This file shows an example of a search strategy for MEDLINE database. (DOCX 47 kb) [file 13643_2017_498_MOESM3_ESM.docx]
