# Supplementary material for: Do outcomes reported in randomised controlled trials of joint replacement surgery fulfil the OMERACT 2.0 Filter? A review of the 2008 and 2013 literature
Source: Syst Rev. 2017 May 30;6:106. doi: 10.1186/s13643-017-0498-3 (PMC5450048; doi:10.1186/s13643-017-0498-3)
Supplement: Supplementary file 4 — Shoulder and Hand Study Outcomes. Description of data: This file shows the included studies for shoulder and hand joint replacement and which of the core areas/domains of OMERACT Filter 2.0 were presented as outcomes in these trials. (DOCX 67 kb) [file 13643_2017_498_MOESM4_ESM.docx]

**Additional File 4.** Shoulder and Hand Study Outcomes

| **Shoulder Studies** | | | | | | | | | | | | | |
| --- | --- | --- | --- | --- | --- | --- | --- | --- | --- | --- | --- | --- | --- |
| **Authors** | **Pathophysiological** | | | | | | | | **Life Impact** | | | **Death** | **Resource Use / Economic impact** |
|  | **Pain** | **ROM** | **Strength** | **Fatty muscle %** | **Healing of bone** | **AE** | **RMAL** | **Stiffness** | **QoL** | **Function** | **Activity levels** | **Mortality** |  |
| **Fialka 2008** | √ | √ | √ |  |  | √ | √ |  |  |  | √ | √ |  |
| **Soliman 2013** | √ | √ | √ |  |  |  |  |  |  |  | √ |  |  |
| **Lapner 2013** | √ |  | √ | √ | √ |  |  |  | √ | √ | √ | √ |  |
| **Hand Studies** | | | | | | | | | | | | | |
| **Hansen 2013** | **√** |  | **√** |  |  |  | √ | √ | √ | √ |  |  |  |
